# Angiomatous Nasal Polyps in a 43-Year-Old Female From North-Central Nigeria: A Case Report

**DOI:** 10.7759/cureus.78506

**Published:** 2025-02-04

**Authors:** Kevin N Ezike, Ijeoma A Okwudire-Ejeh, Kayode A Kamardeen, Olubunmi A Alabi, Bamnan C Dallang, Emmanuel E Oguntebi, Umar M Umar, Muhammed G Mainasara

**Affiliations:** 1 Anatomic Pathology and Forensic Medicine, Nile University of Nigeria, Abuja, NGA; 2 Anatomic Pathology and Forensic Medicine, Asokoro District Hospital, Abuja, NGA; 3 Otolaryngology, Diagnostic and Treatment Center, Central Bank of Nigeria, Abuja, NGA; 4 Obstetrics and Gynecology, Excel Specialist Hospital and Fertility Centre, Abuja, NGA; 5 Radiology, Nile University of Nigeria, Abuja, NGA; 6 Otolaryngology, Kaduna State University, Kaduna, NGA

**Keywords:** angiomatous nasal polyp, chronic rhinosinusitis, nasal congestion, nasal polyps, rhinorrhea

## Abstract

Angiomatous nasal polyps (ANPs) are uncommon variants of nasal polyps, which, like their more common counterparts, are a significant and common cause of morbidity for patients, regardless of demographic variations. As a group, nasal polyps are non-neoplastic and commonly arise within the maxillary antrum, sphenoid, and ethmoid sinuses, protruding via the ostia into the nasal cavity. Their precise cause is unknown, but several factors related to genetics, local infections, family history, and environment have been implicated in their etiopathogenesis. ANPs are commonly diagnosed in the younger age group and are particularly significant because they may mimic benign and malignant vascular tumors occurring within the nasopharynx.

We report a rare case of angiomatous nasal polyp in a 43-year-old Nigerian female. ANPs should be included in the differential diagnosis of mass lesions of the nasal cavity in adult patients (male or female) who present with obstructive symptoms and should be distinguished from both benign and malignant neoplastic mimics.

## Introduction

Nasal polyps are non-neoplastic swellings that usually arise from the middle meatus and ethmoid sinuses and prolapse into the nasal cavity [1]. They are a frequent cause of discomfort and considerable morbidity, afflicting sufferers with symptoms such as nasal obstruction, rhinorrhea, anosmia, and epistaxis [2,3]. The precise pathogenetic mechanism of nasal polyps is unclear but four principal factors - chronic local infection, family history and genetic predisposition, atopy and allergy to inhalant and food allergens, and aerodynamic factors - are considered important in its pathogenesis [4]. Chronic inflammation is considered particularly important, such that nasal polyp is said to be its ultimate manifestation [5].

Four main histological variants of nasal polyps have been described as follows: edematous, eosinophilic (allergic) polyp; fibroinflammatory polyp; polyp with hyperplasia of seromucinous glands; and polyp with stromal atypia [1]. The most common of these, the edematous, eosinophilic (allergic) polyp, accounts for over 85% of cases [1]. Another variant, angiomatous nasal polyp (ANP), considered to be a subvariant of the fibroinflammatory polyps characterized by extensive vascular proliferation and angioectasia, is rarely seen and accounts for 4-5% of all inflammatory nasal polyps [6,7]. Evaluating a patient with suspected nasal polyps involves detailed history taking and careful physical examination. Nasal endoscopy in the clinic and coronal CT scanning of the paranasal sinuses are the tools of choice for diagnosis and determining the extent of disease and possible bone involvement [8]. Medical treatment options abound, but surgery is the mainstay of treatment and the method of choice is endoscopic sinus surgery [8]. We report a rare case of angiomatous nasal polyp in a 43-year-old Nigerian female.

## Case presentation

A 43-year-old female with a history of allergy presented with recurrent bilateral nasal congestion of seven months duration, worse on the right, and associated with rhinorrhea, epiphora, frontal headache, and post-nasal drip. She also had occasional snoring, mouth breathing, recurrent sore throat, aural fullness, and tinnitus.

Examination revealed reduced patency of nasal cavities bilaterally with engorged inferior turbinates. Multiple glistening nasal masses were noted which appeared to arise from the right ethmoidal region. They were non-sensitive and did not bleed on touch.

**Figure 1:**
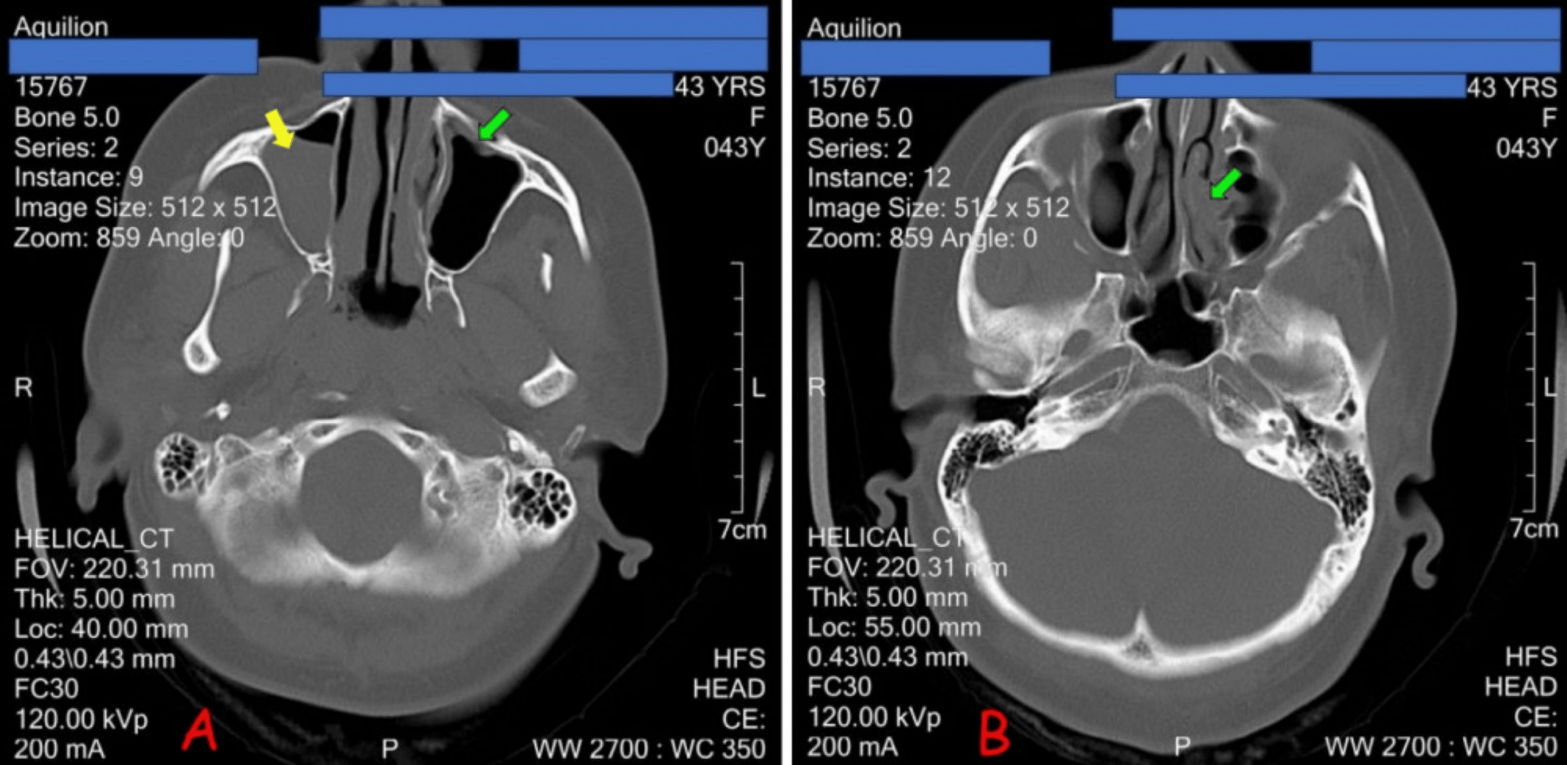
Plain axial CT images of the maxillary sinuses. (A) Isodense mass in the right maxillary sinus (yellow arrow) and increased mucosal thickness in the left maxillary sinus (green arrow). (B) Increased mucosal thickness of the left ethmoidal sinuses (green arrow).

Axial computed tomography (CT) scan of the paranasal sinuses showed a broad-based polypoidal isodense mass in the right maxillary sinus measuring 29.5 x 16.5 mm. No calcification was seen within it. No associated sinus expansion and no bone destruction were seen. There was increased mucosal thickness in the left maxillary and ethmoidal sinuses. Increased mucosal thickness of the nasal turbinates is noted bilaterally (Figures 1A, 1B).

A diagnosis of chronic rhinosinusitis with nasal polyps (CRSwNP) was made and patient was prepared for endoscopic sinus surgery. The procedure was successfully performed under general anesthesia with concurrent nasal topical decongestion using oxymetazoline and 1:50,000 adrenaline for nasal preparation. The patient underwent bilateral uncinectomy, middle meatal antrostomy, anterior ethmoidectomy, and polypectomy. The polypectomy specimens were preserved in 10% neutral buffered formalin and sent for histopathological evaluation.

**Figure 2:**
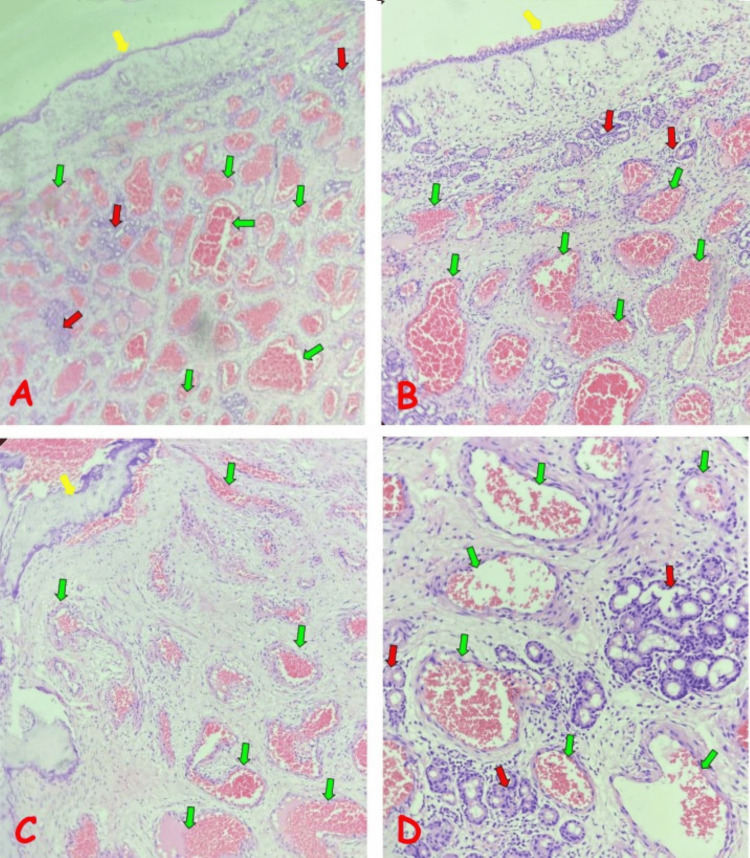
Photomicrographs of angiomatous nasal polyp. (A) Low-power view showing overlying epithelium (yellow arrow), proliferations of variably sized, congested blood vessels (green arrows), and islands of mucous glands (red arrows), all of which are set in fibromyxoid stroma. (B) Medium-power view of the image in (A), with the overlying epithelium (yellow arrow) confirmed to be pseudostratified, ciliated columnar epithelium. Mucous glands (red arrows) are seen in the stroma beneath the epithelium; the endothelium lining the congested blood vessels (green arrows) and the mucinous epithelium lining the mucous glands (red arrows) are also clearly demonstrated. (C) Trabeculae of mature bone at the periphery of the lesion (yellow arrow). The variably sized, congested blood vessels (green arrows) are also shown, set in fibromyxoid stroma. (D) High-power image showing that some of the blood vessels (green arrows) are thick-walled, and there are periglandular infiltrates of chronic inflammatory cells around the mucous glands (red arrows).

Pathological examination revealed gross findings of multiple similar greyish-white polypoid masses with a membranous appearance, together weighing less than 1 g and measuring 2.5 x 2.0 x 0.8 cm, with greyish-white to brownish cut surfaces and focal gritty bony consistency. Microscopic sections showed benign polypoid lesions covered by attenuated, pseudostratified, ciliated columnar epithelium to which trabeculae of mature bone were attached. The underlying stroma was fibromyxoid and contained proliferations of variably sized, frequently congested, thin-walled vascular channels and occasional islands of mucous glands. A scanty chronic inflammatory cells infiltrate was also seen in the stroma. There was no cytological atypia. A diagnosis of angiomatous nasal polyps was made (Figures 2A-2D).

The patient’s post-operative condition was satisfactory, and she was discharged home the next day with instructions on nasal douching, oral antihistamines, analgesics, and antibiotics. Her condition immediately post-surgery, as well as on two subsequent follow-up visits (on days five and 10), was satisfactory, and she reported marked improvement in her symptoms.

## Discussion

Paranasal sinus polyps that protrude into the choana via the sinus ostia are referred to as choanal polyps [6]. Choanal polyps are named according to their site of origin as follows: antrochoanal when they arise from the maxillary antrum, sphenochoanal when they arise from the sphenoid sinus, and ethmoidochoanal when they arise, controversially, from the ethmoid sinus [6]. Due to their origin within a confined space and passage through constrictive ostia, choanal polyps are prone to vascular compromise which sets up a process of vascular dilatation, edema, infarction, and neovascularization culminating in total necrosis or more commonly angiomatous differentiation [6]. The choanal polyp most likely to undergo this transformation is the antrochoanal polyp [3]. In contrast, our patient’s lesions were seen in both the maxillary and ethmoidal sinuses.

ANPs are most commonly diagnosed in children and young adults [3]. However, our 43-year-old patient falls outside this age range. The reason for this predilection for younger patients is unclear, but the likelihood of smaller ostia in the young leading to more severe constriction of the protruding polyp may be a contributing factor. Tam et al. postulate that diabetes mellitus and hypertension may have a role in the development of angiomatous polyps by inducing vascular damage and its sequelae [8]. This postulation is not supported by the fact that ANPs are more commonly seen in younger patients, who are less likely than adults to have diabetes mellitus or hypertension. Furthermore, our patient had no prior history of either condition.

The clinical presentation of ANPs is generally similar to that of other nasal polyps, as they are related to the nasopharynx and cause significant morbidity [4,6]. However, ANPs may also present with epistaxis [3,9]. This tendency to present with epistaxis along with radiological and histological findings places ANPs in the differential diagnosis of vascular tumors of the nasopharynx, including hemangioma and angiofibroma [9].

The definitive management of nasal polyps is surgical but medical management may be used as a short-term remedy or post-surgically to prevent or retard regrowth of the polyps [10,11]. The determinants of treatment modality are influenced by the size of the polyps, severity of symptoms, and associated etiological factors. Inflammatory conditions play a prominent role in the etiology of nasal polyps; therefore, patients benefit from non-specific anti-inflammatory drug therapy, such as the use of oral corticosteroids. Similarly, patients whose polyps are associated with history of environmental allergies may benefit from specific allergy pharmacotherapy [10]. Although our patient had a history of allergy, the severity of her symptoms necessitated recourse to definitive surgical treatment. Surgical treatment of nasal polyps has evolved from antiquity to the present time when endoscopic sinus surgery is the mainstay [11]. Surgery is however contraindicated in patients with severe pulmonary or cardiac problems, bleeding diathesis, and acute asthma exacerbation.

The complications of ANPs may arise either from the lesions themselves, such as severe hemorrhage, bone destruction leading to facial deformities, and proptosis [12], or from their surgical management, which can include cerebrospinal fluid leaks, meningitis, intracranial hemorrhage, brain abscess, brain herniation, blindness, optic nerve injury, orbital hematoma, eye muscle injury leading to diplopia, nasolacrimal duct injury causing epiphora, and vascular injury resulting in severe hemorrhage [11].

In addition to the differential diagnoses already discussed, ANPs may mimic a malignant process by causing bone destruction [7]. Thus, accurate diagnosis through endoscopic and radiological techniques will alleviate patient anxiety and prevent overtreatment.

## Conclusions

ANPs should be included in the differential diagnosis of mass lesions of the nasal cavity in adult patients, male or female, who present with obstructive symptoms. The recognition of the histological features of these lesions is important in order to distinguish them from both benign and malignant neoplastic mimics.
